# Cognitive and sensory expectations independently shape musical expectancy and pleasure

**DOI:** 10.1098/rstb.2022.0420

**Published:** 2024-01-29

**Authors:** Vincent K. M. Cheung, Peter M. C. Harrison, Stefan Koelsch, Marcus T. Pearce, Angela D. Friederici, Lars Meyer

**Affiliations:** ^1^ Sony Computer Science Laboratories, Inc., Shinagawa-ku, Tokyo 141-0022, Japan; ^2^ Max Planck Institute for Human Cognitive and Brain Sciences Ringgold standard institution Leipzig, Sachsen, Germany; ^3^ Institute of Information Science, Academia Sinica, Taipei 115, Taiwan; ^4^ Centre for Music and Science, University of Cambridge, Faculty of Music, 11 West Road, Cambridge, CB3 9DP, UK; ^5^ Centre for Digital Music, Queen Mary University of London, E1 4NS, UK; ^6^ Department of Biological and Medical Psychology, University of Bergen, Bergen, 5009, Norway; ^7^ Department of Clinical Medicine, Aarhus University, Aarhus N, 8200, Denmark; ^8^ Research Group Language Cycles, Max Planck Institute for Human Cognitive and Brain Sciences, Leipzig 04103, Germany; ^9^ Clinic for Phoniatrics and Pedaudiology, University Hospital Münster, Münster, 48149, Germany

**Keywords:** music, tonal harmony, expectancy, pleasure and reward, predictive coding, computational modelling

## Abstract

Expectation is crucial for our enjoyment of music, yet the underlying generative mechanisms remain unclear. While sensory models derive predictions based on local acoustic information in the auditory signal, cognitive models assume abstract knowledge of music structure acquired over the long term. To evaluate these two contrasting mechanisms, we compared simulations from four computational models of musical expectancy against subjective expectancy and pleasantness ratings of over 1000 chords sampled from 739 US Billboard pop songs. Bayesian model comparison revealed that listeners' expectancy and pleasantness ratings were predicted by the independent, non-overlapping, contributions of cognitive and sensory expectations. Furthermore, cognitive expectations explained over twice the variance in listeners’ perceived surprise compared to sensory expectations, suggesting a larger relative importance of long-term representations of music structure over short-term sensory–acoustic information in musical expectancy. Our results thus emphasize the distinct, albeit complementary, roles of cognitive and sensory expectations in shaping musical pleasure, and suggest that this expectancy-driven mechanism depends on musical information represented at different levels of abstraction along the neural hierarchy.

This article is part of the theme issue ‘Art, aesthetics and predictive processing: theoretical and empirical perspectives’.

## 1. Introduction

Music has been an integral part of human culture since prehistoric times [1–3], and most people find music highly rewarding [4,5]. Apart from extra-musical factors such as episodic memory or contextual associations [6,7], an important intra-musical factor by which music itself induces pleasure in the listener is via the confirmation, violation and delay of listeners' musical expectations [8–13]. Here, we consider the hypothesis that expectancy-driven musical pleasure results from two independent sources of expectancy: *sensory expectations* arising from acoustic information in the auditory signal itself, as well as *cognitive expectations* derived from learned relations between musical elements abstracted from the auditory signal. While sensory expectations form over relatively short timescales, cognitive expectations are acquired after extended exposure to multiple examples of a musical style.

One influential neurocognitive model that embodies this view is the predictive coding of music (PCM) model [14–17]. In PCM, musical expectations generated from higher-order brain regions (e.g. prefrontal cortex) are thought to propagate downwards towards lower-level sensory regions (e.g. the auditory cortex). The discrepancy between expected and actual incoming signals in sensory regions results in a prediction error or *surprise*. This error signal is propagated upwards along the cortical hierarchy to refine future expectations. The gain of the expectation is modulated by a precision estimate, or the inverse of *uncertainty*. Precision-weighted prediction error signals are thought to constitute reward for the listener to continue learning towards generating more accurate future expectations [15,18]. In line with PCM, recent evidence has shown that pleasantness ratings of chords and melodies were jointly predicted by their surprise and uncertainty in listeners [19,20]. These studies represented an advancement to previous work examining the link between musical expectancy and pleasure (e.g. [21–23]), as the additional consideration of uncertainty meant that they could explain how musical surprises could be both pleasant and unpleasant. However, these studies only accounted for cognitive expectations, since it was assumed that listeners formed expectations exclusively based on the statistical relationships of chords and pitches in melodies as symbolic entities extracted from the music. The contribution of sensory expectations to musical pleasure thus remained unclear.

### (a) Sensory and cognitive contributions to musical expectancy

Research on sensory and cognitive contributions to musical expectancy has a rich history. Seminal work using the probe-tone method [24–26] established the concept of tonal hierarchy, showing that given a context of tones or chords, the perceived importance of the ensuing tone was related to its proximity with the key—or the tonal centre—of the context. This implies that the structural function of a musical sound is not determined by its absolute frequencies but by its implied relation derived from the context [27], which suggests cognitive contributions to musical expectancy. Similar results were found in subsequent priming studies, which observed a behavioural facilitation when target tones and chords were harmonically more related to the priming chord [28–34] or melody [35,36]. Studies in children [37], adults exposed to a novel musical scale system [38] or style [39], and using cross-cultural designs (see review in [27]) provided further support for a cognitive influence by showing that the tonal hierarchy may be acquired via internalization of regularities between musical elements in a given musical style over extended exposure, in a process known as statistical learning.

While the tonal hierarchy shows that acquired abstract knowledge of music structure guides expectancy, the relative prominence of chords or tones is intrinsically constrained by their acoustic properties. For example, the tonic and the dominant are, respectively, ranked top and second of the tonal hierarchy of Western tonal music [26] but also share highly overlapping harmonic spectra [40]. To overcome this, several studies controlled for the number of shared tones in the priming context and target [31,33], manipulated the stimulus onset asynchrony [41], or presented stimuli using piano and pure-tone timbres [35]. Interestingly, a facilitation in processing harmonically more-related over less-related targets was still observed, except when the priming context was presented very rapidly (75 ms per chord) and was previously unheard by the subject [31]. Although these studies suggest the dominant influence of cognitive over sensory information in forming expectations during music-listening, the underlying mechanisms could only be inferred from manipulations in the study design.

In recent years, computational models of musical expectancy have been devised to provide an algorithmic formalization of how musical expectations could be generated [10,42]. Computational models of expectancy can be placed along a sensory–cognitive continuum, depending on the extent to which sensory or cognitive information is given prominence when forming expectations [43]. As underlying hypotheses and assumptions are formalized and made explicit, the comparison of simulations from computational models with behavioural data provides a direct assessment of the plausibility of the biological or cognitive mechanisms embodied by each model [10,44]. Such an approach has been used to show that sensory expectations as simulated by an auditory short-term memory model could explain many of the priming effects previously taken to support cognitive accounts—even when sensory influences have been accounted for [40,45]. Other studies paint a more nuanced picture, with the comparison of simulations from several computational models against behavioural data generally supporting the influence of cognitive over sensory mechanisms [34,43,46–49]. Furthermore, a recent comparison of melodic expectations showed that low-level (although not explicitly sensory) and cognitive information may explain non-overlapping behavioural variance [48]. This is consistent with the hierarchical representation of prediction errors in the PCM model and is in line with priming studies showing cognitive and sensory facilitation at different timescales [31,41]. However, the interpretation of these existing results is complicated by their use of only weakly sensory or weakly cognitive models applied to restricted stimulus domains. Therefore, it remains to be clarified whether sensory and cognitive influences independently contribute to musical expectancy, or whether they have an interactive effect, suggesting a combined underlying mechanism [48].

### (b) The current study

Here, we tested the ways in which cognitive and sensory information contribute towards harmonic expectations and musical pleasure in listeners encultured to Western tonal music. Our approach was to compare ratings of listeners’ continuous chord surprise (Experiment 1, §2a(i)) and pleasantness (Experiment 2, §2a(ii)) ratings against simulations from cognitive and sensory computational models of musical expectancy. Subjects were presented with 30 isochronous chord progressions sampled from commercially successful pop songs in the McGill Billboard dataset [50], which contains over 80 000 chords from 745 pop songs listed on the US Billboard ‘Hot 100′ chart between 1958 and 1991. Apart from enabling a direct investigation on the underlying generative mechanisms, the parametrical quantification of expectancy with computational models also allowed us to present stimuli derived from actual music. This is an alternative to the traditional approach of using carefully constructed artificial stimuli to isolate sensory and cognitive influences, in which stimuli represent a small number of categories of expectation (e.g. expected and unexpected, sometimes supplemented by an intermediate category) and are often repeated many times [19,51].

Whereas our previous work [19] showed that temporal states of expectancy—*cognitive* uncertainty and surprise—jointly modulated listeners' pleasantness ratings, the current study investigated how and to what extent cognitive *and* sensory information shape musical expectancy and pleasure. We further tested the role of musical training in cognitive and sensory contributions to chord expectations by acquiring data from musicians and non-musicians in Experiment 1. With increased musical exposure and knowledge, we expected increased cognitive relative to sensory influence on chord surprise ratings in musicians compared to non-musicians.

### (c) Computational models of musical expectancy

We considered four representative computational models of musical expectancy that span the sensory–cognitive continuum. The first two models can be regarded as purely sensory models because they derive chord expectations based on processing acoustic information. The third model can be thought of as a hybrid sensory–cognitive model because it assumes both sensory and (limited) cognitive contributions in simulating expectancy from the auditory signal input. The last model can be considered as a purely cognitive model because it operates completely on the symbolic level and presupposes the abstraction of chord representations from the auditory signal by the listener.

#### (i) Spectral distance

The spectral distance (SD) model [52,53] computes expectancy in terms of spectral similarity between two adjacent chords. Each chord is modelled as a linear combination of 12 harmonic overtones, and each overtone is represented as a one-hot vector encoding its pitch or pitch class on a log-frequency scale. A weighting function is then applied to discount perceptual salience at higher overtones, followed by Gaussian smoothing to account for perceptual noise. The 12 overtone vectors are then summed to produce the spectral vector of the chord. SD is obtained by taking the cosine distance of spectral vectors of two adjacent chords, with 1 being completely orthogonal and 0 being perfectly correlated. Chords perceived by the listener as surprising would be expected to show high SD.

#### (ii) Periodicity pitch

The periodicity pitch (PP) model [54] is a psychoacoustic model that simulates expectancy based on neural encoding in the peripheral auditory system. The auditory signal is first transduced into simulated firing patterns of inner-hair cells in the cochlea that encode PP information. Next, firing patterns are transformed into so-called *pitch images* by bandpass filtering and taking a windowed autocorrelation. To simulate exponential decay in auditory short-term memory, the pitch images are then filtered at two different timescales to generate a global and a local pitch image. The global pitch image has a longer integration time and represents the contextual information held in echoic memory, while the local pitch image has a shorter integration time and reflects the perception of the immediate chord. The tonal contextuality is then computed by evaluating the *z*-transformed Pearson's correlation between global and local pitch images. This measure reflects the similarity between the incoming chord and its preceding context within the memory trace. For ease of comparison, we multiply tonal contextuality by −1 to obtain a quantity referred to as tonal dissimilarity. We would expect chords perceived as surprising to show high tonal dissimilarity.

#### (iii) Tonal expectation

The tonal expectation (TE) model [43] extends the PP model by hypothesizing that acoustic features present in the auditory input are processed and represented at the sensory and cognitive levels. Apart from representing PP information in echoic memory as in the original model, two additional representations are introduced. First, a chroma vector representation is derived by assigning PP representations into discrete pitch classes to account for the perceptual phenomenon of octave equivalence. Second, a tonal space representation is obtained by mapping the PP representation onto the space spanned by the tonal hierarchy. As the tonal hierarchy assumes abstract knowledge of music structure acquired via statistical learning in the listener, a cognitive contribution to chord expectancy is modelled.

Similar to the PP model, information about the chord context and the immediate chord is summarized by global and local pitch images computed at the three levels of representation. Chord expectancy is derived by taking a weighted sum of the tonal contextuality and the maximum value of the global pitch images for each representation. The weights are determined *a priori* using stepwise regression to best fit reaction time differences between harmonically related and unrelated targets from seven priming studies. Chords perceived as surprising are expected to be processed with a slower reaction time by this model.

#### (iv) Information dynamics of music

The information dynamics of music (IDyOM) model [55,56] is a computational model of expectancy embodying two hypotheses: first, that listeners acquire internal cognitive representations of regularities in a musical style through statistical learning of the music to which they are exposed; second, that musical expectations reflect probabilistic predictions for forthcoming musical events derived from these internal representations. IDyOM prospectively generates probability distributions for each event in a piece of music, conditional on the preceding musical context (intended to simulate online learning during stimulus presentation) and the prior musical experience of the model (intended to simulate the listener's prior long-term musical exposure).

Two information-theoretic measures of expectancy are distinguished by IDyOM. First, the surprise elicited by a musical event is quantified by its information content (IC), or negative log-probability. IC reflects the extent to which the model did not expect an event given the particular context in which it appeared. Chords with higher IC are hypothesized to be rated as more surprising. Second, the uncertainty about the event is quantified as the entropy of the distribution. Maximum entropy occurs when all possible continuations are equiprobable given the context. Note that for our comparison between chord surprise ratings and model simulations in Experiment 1, only the IC of each chord is considered from this model. Both IC and entropy are used to predict chord pleasantness in Experiment 2. To simulate the long-term exposure of a listener to Western pop music, we trained IDyOM on all chords from the McGill Billboard dataset [50].

## 2. Methods and materials

### (a) Subjects

#### (i) Experiment 1

Twenty-five healthy adults (13 musicians and 12 non-musicians) participated in Experiment 1. Musicians (mean age = 24.4 y, s.d. = 3.48) scored a mean of 40.5 (s.d. = 4.41) in the musical training subscale of Goldsmiths Musical Sophistication Index (Gold-MSI) [57], while non-musicians (mean age = 26.1 y, s.d. = 4.56) scored a mean of 12.5 (s.d. = 5.64). These, respectively, corresponded to the 86th and 15th percentile scores from self-recruited participants for the online ‘How Musical are You’ BBC study [57]. The two groups thus showed differences in musical training (Wilcoxon's rank sum: *W* = 156) but not in age (Welch's *t*: *t*_20.5_ = −1.04). Our sample size was justified based on power calculations. Assuming a power of 0.8 and a two-sided significance level of 0.05, the relationship between behavioural measures of expectancy and simulations from multiple computational models (including those not considered in the present study) as reported in [34,47,52] yielded an estimated minimum sample size of 25 subjects.

Inclusion criteria for musicians were musical training (in addition to music lessons at school) at or before age 10, at least seven continuous years of musical training and actively practicing their instruments on average at least once a week for the past three years. Inclusion criteria for non-musicians were no musical training before age 7, no musical training in the past 7 years and less than 10 years of continuous musical training in total. An exclusion criterion for both groups was absolute-pitch, for which subjects were screened prior to the experiment using the absolute-pitch test devised in [58]. Additional data from one non-musician were not analysed due to non-compliance with the experimental procedure. All subjects reported right-handedness and normal hearing. Data from this experiment were collected specifically for this study and have not been published before.

#### (ii) Experiment 2

We analysed data from 39 healthy adults with no restriction on musical training (mean age = 24.1 y, s.d. = 3.80). Subjects scored a mean of 23.4 (s.d. = 10.3) on the musical training subscale of the Gold-MSI, which corresponded to the 40th percentile score. One subject was excluded as they did not comply with the experimental procedure. These data were analysed in our previous study [19] that examined the role of uncertainty and surprise in musical pleasure and brain activity. As the joint role of uncertainty and surprise on musical pleasure was essentially unexplored prior to that study, we aimed for a sample size that was comparable to previous studies using IDyOM to investigate effects of uncertainty (e.g. [49] with 30 subjects) or surprise (e.g. [34] with 40 subjects, and [47] with 50 subjects) during music perception.

Our study was approved by the Ethical Committee of the Medical Faculty at Leipzig University and written informed consent was obtained from all subjects. This study was not preregistered.

### (b) Stimuli

Subjects were auditorily presented with 30 isochronous chord progressions taken from commercially successful pop songs listed in the McGill Billboard dataset [50]. These stimuli were identical to those from our previous study that tested the effects of expectancy on musical pleasure and brain activity [19]. Each chord progression ranged between 30 and 38 chords inclusive (mean number of chords = 34.6, s.d. = 2.17) and the duration of each chord was 2.4 s. To mitigate confounding effects from interactions with other dimensions (such as timing and language) and familiarity, only the chord progression of the original song was retained and transposed to C major. Each chord was computer-generated with a timbre synthesized from a marimba, jazz guitar and an acoustic guitar. All timbres played all pitches in each chord. Damping and reverb were adjusted to ensure no spill-over to the next chord. To give momentum to the stimuli, each chord progression was also accompanied by one of three background rhythms that repeated once per chord. The assignment of background rhythms to each stimulus was counterbalanced across subjects.

### (c) Computational model parameters

SD: Taking the optimized parameters as given in [53], we used a weighting of *i*^−0.75^ for the *i*th harmonic overtone, where *i* = 1,…,12, a pitch-class representation and a smoothing parameter of 6.83. A Matlab implementation can be found at the following page: http://www.dynamictonality.com/probe_tone_files.

PP: Although time constants 1.5 s for the global pitch image and 0.1 s for the local pitch image were originally used as they best fitted the probe-tone data of [26], following the approach of [40], we explored other parameter combinations. We tested six different model combinations with global pitch image decay constants of 1.5 s (default), 2.5 s and 4 s (to match the span of echoic memory [34,59]) and local pitch image decay constants of 0.1 s (default) and 0.5 s. Comparing the expected log pointwise predictive density (ELPD) (see §2e) for the six model combinations, we selected the model with global and pitch image constants of 4 s and 0.5 s in our experimental analyses as it showed the best out-of-sample predictive accuracy (electronic supplementary material, figure S1). Matlab code for the model can be accessed at: https://github.com/IPEM/IPEMToolbox.

TE: We used the default parameters of the model as optimized for seven probe tone and priming studies. A Matlab implementation is available at https://atonal.ucdavis.edu/resources/software/jlmt/.

IDyOM: IDyOM is a variable-order Markov model that generates the conditional probability of a chord given previous chords in a progression by combining its *n*-gram models of different orders (i.e. different values of *n*) using the PPM* algorithm [60–62]. Given a chord progression $\left\{e_{1},\ldots ,e_{i},\ldots \;,e_{j}\right\}$, the *n*-gram probability of chord $e_{i}$ is expressed as $p(e_{i}\;|\;e_{i-(n-1)}\;,\ldots \;,e_{i-1})$. There are two sub-components to IDyOM: in the long-term model (LTM), the conditional probability is estimated from all chord sub-sequences in the training set, while in the short-term model (STM), the conditional probability is estimated from all previously observed sub-sequences in the current song. The LTM and STM probabilities are then combined by taking the geometric mean (which provides better prediction performance than an arithmetic mean [55]). We applied 10-fold cross-validation when training the model on the McGill Billboard dataset to prevent overfitting to specific songs (and so that the presented stimuli were not part of the training data) and selected a maximum *n*-gram order of 10 in the LTM and unbounded in the STM. Although IDyOM could be extended to incorporate surface features such as voicing, note duration and pitch interval, we do not model these multiple viewpoints here for parsimony and to ensure that the model simulations are not influenced by any possible sensory–acoustic information.

IDyOM simulates the surprise of a chord by quantifying its IC, which is an information-theoretic measure of the amount of information transferred when a signal is sampled. The IC of a chord is given by its negative log-probability conditional on the context of preceding chords, i.e. $IC(e_{i})=\;-\log_{2}p(e_{i}\;|\;e_{i-(n-1)}\;,\ldots \;,e_{i-1})$. Chords that appear rarely in a given context will have a high IC, and so we expect that chords with higher IC will be perceived by the listeners as more surprising.

On the other hand, IDyOM simulates the uncertainty of chord by quantifying its entropy *H*, which is obtained by summing the product between the conditional probability of all possible chords in *S* in the training corpus and their ICs, i.e.

$$H(e_{i})=-\underset{e\in S}{\sum }p(e_{i}=e|\;e_{i-(n-1)}\;,\ldots ,e_{i-1})\log_{2}p(e_{i}=e|\;e_{i-(n-1)}\;,\ldots ,e_{i-1}).$$

Entropy therefore provides a measure for the average surprise of a chord at that position in the progression, given its preceding context. Previous work has shown that listeners regard notes in melodies with higher entropy as more uncertain [49]. Code for the IDyOM project can be found at: https://github.com/mtpearce/idyom.

### (d) Procedure

During stimulus presentation, subjects gave continuous ratings of their perceived surprise (Experiment 1) or pleasantness (Experiment 2) for each chord using a 10 cm mechanical slider analogous to our previous experiment [19]. This allowed us to acquire observations without restriction to a particular point in the stimulus (see e.g. [63] on potential closure effects) and to present stimuli with a much longer context (at least 30 chords in each progression compared to approx. 10 chords in previous studies [40,43,47]) to match actual music more closely.

Subjects were asked to hold the slider with their left hand and to place it on their lap while controlling the slider knob with their right thumb. At the start of each trial, subjects moved the slider to the lowest position and stimulus presentation began 2 s later. They were asked to rate how surprised they were by each chord (Experiment 1) or how pleasant they found each chord (Experiment 2), based on the chords they had heard so far in the stimulus by moving the slider knob to its appropriate position. A rating away from the body indicated higher surprise or pleasure (with maxima indicating ‘very surprised’ or ‘very pleasant’) and closer to the body lower (with minima indicating ‘not surprised’ or ‘not pleasant’). They were prompted to use the entire length of the slider and to give at least five extremal ratings throughout the entire experiment. For each rating, the distance between the slider knob and the minima was discretized linearly to a value between 0 and 1023 (inclusive). This continuous rating procedure allowed us to acquire observations without restriction to a particular chord within the progression (which could lead to potential closure effects [63] or preparatory artefacts [51]), as well as to present stimuli with a much longer context compared to previous studies (at least 30 chords in each progression compared to approximately 10 chords in priming studies). After each stimulus presentation, subjects rated their overall valence and arousal to the stimulus each within a 3 s time window using a 1–6 scale on the keyboard. The next trial then ensued as before.

The presentation of the 30 chord progressions (with 10 assigned to each of the three background rhythms) was pseudo-randomized. The experiment was conducted in a soundproof cabin and stimuli were delivered using supra-aural headphones (Beyerdynamic DT 770 PRO) at a comfortable volume using Psychtoolbox 3 [64] in Octave 4.0.0 [65]. The slider interfaced with the computer using an Arduino Micro sampling at 20 Hz. Prior to the experiment, subjects practised on three trials with a stimulus set different from the actual stimuli. After the experiment, they were asked if the song and artist of our chord stimuli could be identified. No relevant artists or songs were named.

### (e) Statistical analyses

We first preprocessed the behavioural data by applying a five-sample symmetric median filter to the slider responses for each subject to remove small signal fluctuations. The mode of the response was then sampled for each chord in the stimuli in the time window between 1 s after its onset and start of the subsequent chord. This time window was chosen to account for the delayed reaction in moving the slider when hearing a new chord. Ratings for the first chord of each stimulus were discarded as each trial began with the subject resetting the slider. We then mean-averaged each chord rating across subjects within musicians and non-musicians in Experiment 1, and across all subjects in Experiment 2. This resulted in 1009 × 2 = 2018 chord surprise ratings in Experiment 1 and 1009 chord pleasantness ratings in Experiment 2. Stationarity was assessed with the Augmented Dickey Fuller test [66] and by examining autocorrelation and partial autocorrelation functions, which suggested that differencing was not required.

We then modelled the relationship between computational model simulations and behavioural chord surprise and pleasantness ratings with Bayesian multilevel linear models. All variables were first standardized to reduce collinearity and so that effect sizes were on a comparable scale.

In Experiment 1, we first fitted a null model as baseline. This null model comprised a varying intercept by song, and varying slopes (by song) for musicianship (binary-coded), valence and arousal. Varying intercepts and slopes account for differential effects across the stimuli and thus improve parameter estimation by partial pooling of information [67]. We further imposed an AR(1) covariance structure on the chord order of each stimulus to account for temporal autocorrelations in the slider ratings. Although the ‘maximal approach’ [68] also suggests the inclusion of random effects grouped by subject (i.e. not averaging across subjects in each group), we did not do so here as the data required differencing due to non-stationarity and the resulting model fit was poor, with residuals becoming severely heteroscedastic, and thus violated the assumption of linear regression.

Next, for each computational model, we added its simulation and its interaction with musicianship as additional varying slopes to the null model. This allowed us to separately model the relationship between computational model simulations and surprise ratings by musicians and non-musicians.

To test for the additive contribution of IDyOM and PP, we added varying slopes for IC, IC x musicianship, tonal dissimilarity, and tonal dissimilarity x musicianship to the null model. To test for the supra-additive contribution of IDyOM and PP, we further included varying slopes for IC x tonal dissimilarity and IC x tonal dissimilarity x musicianship to the additive model.

In Experiment 2, all models comprised a varying intercept by song, and varying slopes (by song) for valence, arousal, dissonance, spectral centroid and spectral complexity. Please note that the latter three predictors were also included in the model of our original study to control for acoustic differences between each chord, and they are not the same as the sensory expectancy derived from acoustic information as given by PP. To predict chord pleasantness with expectancy simulations by IDyOM as in our original work, we further included IC, entropy and IC x entropy as varying slopes. Likewise, for PP, we included a varying slope for tonal dissimilarity. To test additive effects between simulations by PP and IDyOM, we included varying slopes for tonal dissimilarity, IC, entropy and IC x entropy. To test for supra-additive effects, we further included varying slopes for tonal dissimilarity x IC x entropy, tonal dissimilarity x IC and tonal dissimilarity x entropy from the additive model.

For all models, we used a Gaussian likelihood and weakly informative priors for regularization as previously suggested [67]: Normal(0,1) for parameter estimates, Half-Cauchy(0,1) for scale parameters, and LKJ(2) for the covariance matrix. Inference on the linear relationship between simulation and behaviour was made by examining the parameter estimate of the posterior distribution. We also compared each Bayesian multilevel model using the ELPD derived from Pareto-smoothed importance sampling leave-one-out cross-validation (PSIS-LOO) [69] using the loo package (version 2.2.0; see https://cran.rproject.org/web/packages/loo/). This measure integrates over the posterior distribution of each model to estimate its out-of-sample predictive accuracy, so that models with a better fit to held-out data will have higher ELPD values. More precisely, for observations $\left\{y_{1},\ldots ,y_{N}\right\}$, posterior samples $\left\{\theta^{1},\ldots ,\theta^{S}\right\}$ and truncated Pareto-smoothed importance weights $w_{n}^{s}$ on held-out data *n* and posterior draw *s*, the estimated ELPD is given by:

$$\hat{\mathrm{ELPD}}=\sum_{n=1}^{N}\log\left(\frac{\sum_{s=1}^{S}w_{n}^{s}\;p(y_{n}|\theta^{S})}{\sum_{s=1}^{S}w_{n}^{s}\;}\right).$$

The computation of ELPD allows us to use Bayesian stacking to find the optimal weight of each computational model that jointly maximizes the ELPD of the combined predictive distribution to maximize predictive accuracy.

The joint posterior distribution of model parameters was sampled using adaptive Hamiltonian Monte Carlo in RStan 2.18.2 (Stan Development Team RStan; see https://cran.rproject.org/web/packages/rstan/) using brms 2.9.0 [70] in R 3.5.3 [71]) with four Markov chains (2500 iterations for warm-up, 2500 iterations for sampling) resulting in a total of 10000 samples. Convergence of the Markov chains was examined by visual inspection of parameter trace plots and R-hat values, with no evidence of non-convergence. Model fit was further assessed by comparing simulated and actual responses through posterior predictive checks.

## 3. Results

### (a) Experiment 1: Simulating chord surprise ratings with sensory and cognitive computational models

#### (i) Comparing chord expectancy simulations across computational models

**Figure 1.**
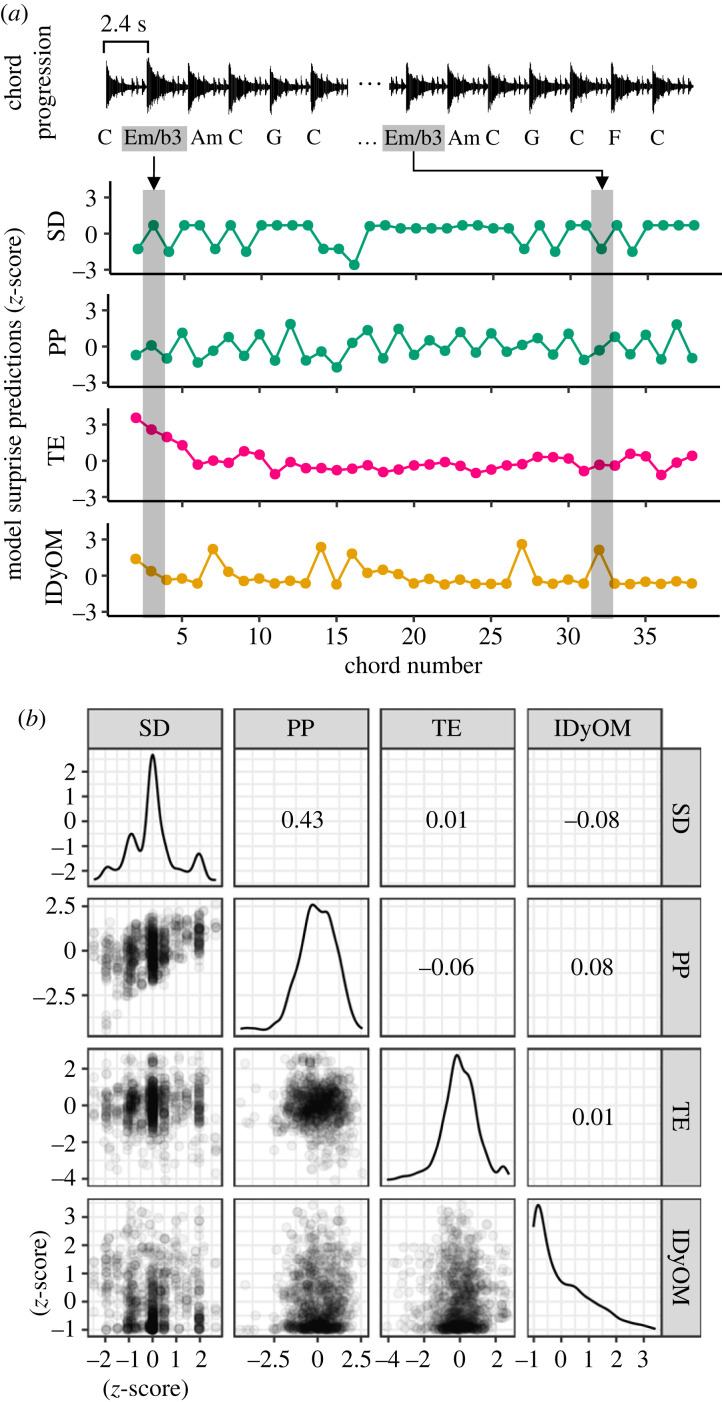
Evaluating cognitive and sensory contributions to chord expectancy. (*a*) Four computational models of music expectancy along a sensory–cognitive continuum were employed to simulate the surprise of a listener to chords in a progression (here, ‘Ob-La-Di-Ob-La-Da’ by The Beatles; refer to electronic supplementary material, audio S1). As shown in the grey bars, the computational model may simulate a different level of surprise for the same chord due to the preceding musical context. (*b*) Scatterplot and marginal densities of the simulated surprise by the four computational models of all chords (*n* = 1039) in the presented stimuli. Computational model abbreviations: SD, spectral distance, PP, periodicity pitch; TE, tonal expectation; IDyOM, information dynamics of music.

We first compared the simulated chord surprise of our stimuli—chord progressions derived from commercially successful pop songs—across the four computational models of musical expectancy. Figure 1 shows the simulated expectancy as given by each model against actual behavioural ratings for one stimulus, as well as their pairwise relationships across all stimuli. We note that although simulations given by the two sensory models—PP and SD—were similar, with a Pearson correlation of 0.43, the remaining pairwise correlations were low and thus indicated a weak relationship between the predictions of the different models. The highly left-skewed density plot for the IDyOM model moreover suggests that our stimuli were dominated by chords with low IC except for a few chords with high IC. By contrast, density plots for PP, SD and the TE model reveal that the majority of chords were predicted to evoke an average level of sensory surprise.

#### (ii) Relating chord surprise ratings with computational model simulations

Next, we built Bayesian multilevel regression models to model listeners' chord surprise ratings. As baseline, we estimated a null model that included a varying intercept by song, a varying slope (by song) for musicianship and varying slopes for valence and arousal ratings. This allowed us to estimate the mean surprise rating given by musicians and non-musicians across all stimuli, after controlling for effects of valence and arousal and idiosyncrasies of each chord progression. On average, musicians tended to give lower chord surprise ratings than non-musicians (difference: *β* = –0.280, 95%-credible interval (CrI) = [–0.453, –0.106]). Increased arousal was also indicative of higher surprise (*β* = 0.177, 95%CrI = [0.025, 0.324]), but the effect of valence (*β* = 0.029) was inconclusive given the inclusion of zero in the 95%-credible interval ([–0.113, 0.174]).

**Figure 2.**
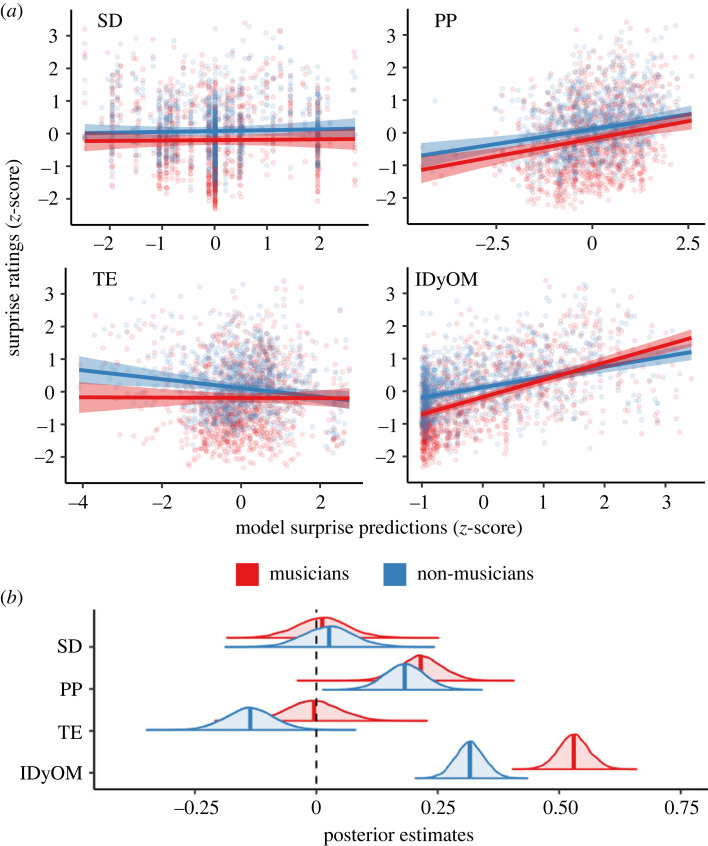
Explaining behavioural surprise ratings with computational model simulations. (*a*) Musicians and non-musicians gave continuous ratings of how surprising they found 1039 chords in 30 isochronous chord progressions sampled from commercially successful pop songs. Bayesian multilevel linear models were fitted and compared to test the relationship between computational model simulations and chord surprise ratings. A substantial positive relationship was found between chord surprise and simulations by PP and IDyOM, with the latter showing a stronger association in musicians compared to non-musicians. By contrast, no meaningful relationship was found for TE and SD. Solid lines indicate the mean posterior estimate. Shaded regions depict the 95%-credible interval of the mean. Filled circles show mean behavioural ratings averaged within the two groups. (*b*) Posterior densities of the effect, separately for musicians (red) and non-musicians (blue). Vertical line indicates the mean of the density, shaded region indicates 95%-credible interval of the mean.

To model the linear relationship between behavioural surprise ratings and computational models’ expectancy simulations, we added predictions from each computational model to the null model, yielding four additional regression models (i.e. one per computational model). We found a clear positive relationship between chord surprise ratings and expectancy simulations by IDyOM and PP, as shown in figure 2. For IDyOM, a one standard deviation-increase in chord IC was related to a 0.530 standard deviation-increase in chord surprise ratings in musicians (95%CrI = [0.469, 0.592]) and a 0.316 standard deviation-increase in non-musicians (95%CrI = [0.257, 0.375]), and the difference between musicians and non-musicians was substantial (*β* = 0.214, 95%CrI = [0.158, 0.272]). For PP, the standardized effect sizes were likewise higher in musicians compared to non-musicians (*β* = 0.215, 95%CrI = [0.127, 0.302], and *β* = 0.182, 95%CrI = [0.098, 0.265], respectively), although evidence for this difference was marginal (*β* = 0.034, 95%CrI = [–0.370, 0.104]). By contrast, no meaningful associations were found between expectancy simulations by SD and surprise ratings by musicians and non-musicians (*β* = 0.012, 95%CrI = [–0.094, 0.122] and *β* = 0.026, 95%CrI = [–0.077, 0.135], respectively). Furthermore, contrary to our expectation, a substantial *negative* association was detected between expectancy simulations by TE and ratings by non-musicians (*β* = –0.136, 95%CrI = [–0.231, –0.037]), although this relationship was not evidenced in musicians (*β* = –0.005, 95%CrI = [–0.108, 0.101]). The relationship between surprise ratings and simulations by SD and TE remained unchanged even after varying slopes for valence and arousal were removed from the models (electronic supplementary material, figure S2), suggesting that the lack of a meaningful association was not due to valence or arousal confounds.

#### (iii) Assessing predictive accuracy of computational model simulations

Apart from examining the strength of the relationship between subjective ratings and model simulations of surprise, an alternative approach is to evaluate each computational model's ability to generalize and accurately simulate surprise ratings from chords in a progression that it had not encountered before. To this end, we computed the predictive accuracy of each model using Pareto-smoothed importance-sampling leave-one-out cross-validation (PSIS-LOO), and compared their relative predictive accuracy in terms of differences in expected log pointwise predictive density (dELPD).

**Figure 3.**
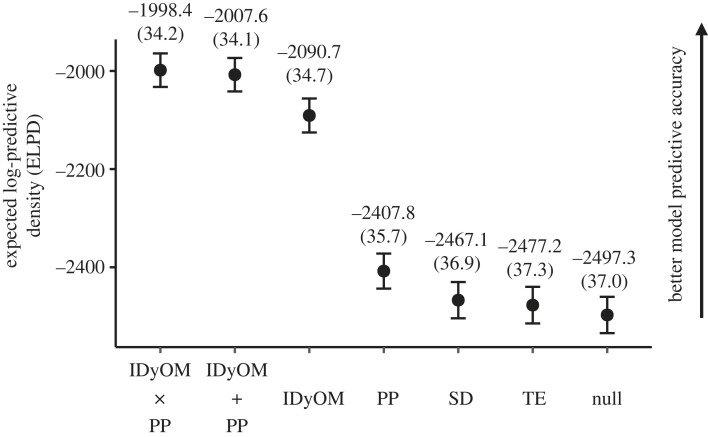
Comparing predictive accuracy of Bayesian multilevel regression models incorporating surprise simulations from computational models using leave-one-out cross-validation. The best model included simulations from both PP and IDyOM in an additive manner, further suggesting unique cognitive and sensory contributions to musical expectancy. Filled circles and error bars, as well as the numbers above and in brackets, respectively, denote the expected log pointwise predictive density (ELPD) and its standard error.

As shown in figure 3, among the four computational model simulations and the null model, the model incorporating chord IC by IDyOM delivered the highest expected out-of-sample performance and exceeded other models by a considerable margin. Notably, the difference in predictive accuracy between IDyOM and the null model (dELPD = 406.5, distance standard error (dSE) = 28.2) was over four times greater than that between PP in second place and the null model (dELPD = 89.4, dSE = 14.9). Including expectancy simulations by SD and TE to the null model also improved predictive accuracy, although gains were more modest (dELPD = 30.2, dSE = 9.1, and dELPD = 20.1, dSE = 7.1, respectively). This suggests that chord expectancy simulations by all four computational models can—each to a different extent—improve predictive performance compared to simply modelling the average surprise rating in musicians and non-musicians.

Next, we used Bayesian stacking to identify the relative contribution of each computational model that would maximize predictive accuracy from a weighted-average of their predictions. Interestingly, most of the weight (90.9%) was assigned to the null model incorporating simulations by IDyOM only, and all remaining weight (9.1%) was assigned to the null model incorporating simulations by PP only. This suggests that a better predictive performance could be attained by combining expectancy simulations from both IDyOM and PP—with a larger contribution from the former—rather than relying on either model alone. Furthermore, although including simulations by SD or TE to the null model improved performance, the fact that they received zero stacking weights suggests that the models were slightly overfit. This interpretation is consistent with the observation above that associations between chord surprise ratings and expectancy simulations by SD and TE were mostly weak or contrary to expectation.

#### (iv) Information content and tonal dissimilarity independently explain chord surprise ratings

The results thus far offer considerable support for IDyOM and PP as computational models that accurately simulate listeners' chord surprise ratings. However, it remained unclear whether IC as provided by IDyOM and tonal dissimilarity as given by PP explain unique variance. To this end, we built two new models: first, a null model with both information content (IDyOM) and tonal dissimilarity (PP) included to test for additive effects; second, another that further included their interaction to test for supra-additive effects.

**Figure 4.**
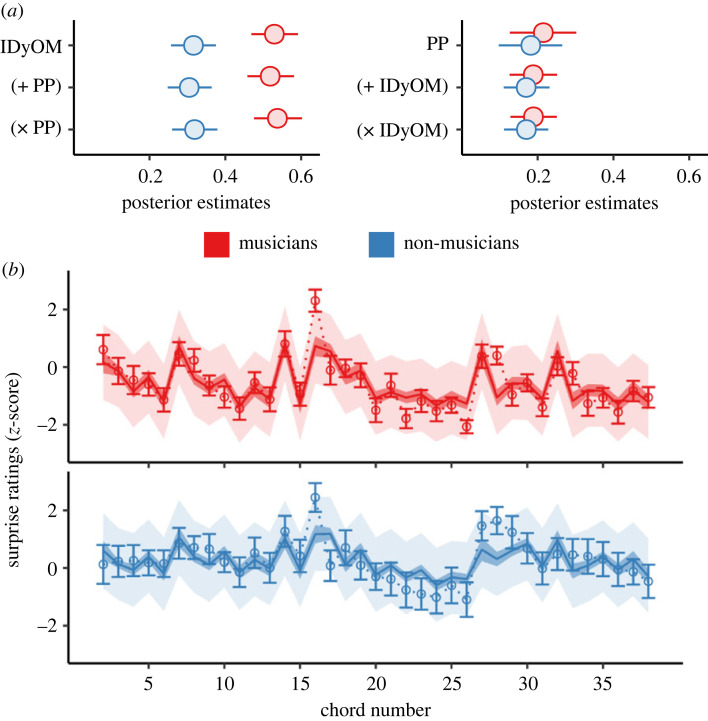
Independent sensory and cognitive contributions to chord surprise. (*a*) Posterior estimates (mean and 95%-credible interval) of information content (as simulated by IDyOM) and tonal dissimilarity (as simulated by PP) on predicting chord surprise ratings when considered separately, additively and supra-additively across musicians and non-musicians. That each effect remained stable when considered individually or together indicates that the two models each explained unique behavioural variation. (*b*) Comparing observed and simulated chord surprise ratings in the additive model. Solid lines indicate the mean predicted response of each model to a chord progression taken from ‘Ob-La-Di-Ob-La-Da’ by The Beatles. Darker shaded regions show the 95%-credible interval of the mean. Lighter shaded regions show the 95%-credible interval of predicted responses. Open circles indicate actual mean behavioural response averaged within the two groups. Error bars depict standard error of the mean. Please see figure 5*c* for the chord names of this stimulus. (Online version in colour.)

As shown in figure 4, the standardized effect sizes and uncertainty estimates for IC and tonal dissimilarity remained virtually unchanged regardless of the presence of the other variable. As before, the positive association between chord surprise ratings and IC was stronger for musicians compared to non-musicians (additive model: musicians *β* = 0.519, 95%CrI = [0.459, 0.582], non-musicians *β* = 0.305, 95%CrI = [0.248, 0.364], difference *β* = 0.214, 95%CrI = [0.160, 0.270]; supra-additive model: musicians *β* = 0.539, 95%CrI = [0.476, 0.604], non-musicians *β* = 0.320, 95%CrI = [0.260, 0.381], difference *β* = 0.219, 95%CrI = [0.164, 0.276]). Likewise, we did not detect a meaningful difference in the positive association between surprise and tonal dissimilarity (additive model: musicians *β* = 0.188, 95%CrI = [0.126, 0.251], non-musicians *β* = 0.170, 95%CrI = [0.111, 0.231], difference *β* = 0.019, 95%CrI = [–0.039, 0.076]; supra-additive model: musicians *β* = 0.189, 95%CrI = [0.127, 0.250], non-musicians *β* = 0.171, 95%CrI = [0.113, 0.228], difference *β* = 0.018, 95%CrI = [–0.040, 0.075]). Notably, the amount of variance explained by IC was approximately three times that of tonal dissimilarity in musicians, and twice that in non-musicians. Furthermore, there was no evidence for a supra-additive effect of IC and tonal dissimilarity (musicians *β* = 0.018, 95%CrI = [–0.049, 0.085], non-musicians *β* = 0.030, 95%CrI = [–0.035, 0.093]).

Similar conclusions are reached from a model comparisons perspective (figure 3). The highest relative predictive accuracy was attained by the supra-additive model, although the improvement in performance with the additive model was marginal and within two-standard errors (dELPD = 9.2, dSE = 5.2). This suggests that the two models gave very similar predictions, and implies that the interaction effects between IC and tonal dissimilarity were likely redundant. We nevertheless observed substantial gains in predictive accuracy in the additive model compared to models incorporating only one of IC or tonal dissimilarity (dELPD = 83.1, dSE = 13.3, and dELPD = 400.2, dSE = 28.4, respectively). Taken together, these results strongly support the proposition that simulations by IDyOM and PP each explain distinct, non-overlapping behavioural variance. This suggests an independent contribution of cognitive and sensory information towards listeners’ expectations of chords.

### (b) Experiment 2: Predicting listeners' pleasantness ratings with chord expectancy simulations from sensory and cognitive computational models

In our previous work [19], we showed that chord expectancy simulations from IDyOM—quantified in terms of IC and entropy—could reliably predict listeners’ pleasantness ratings to the same chord progression stimuli we presented in Experiment 1. Although this provided evidence supporting the role of expectancy in shaping musical pleasure, it is limited as only cognitive expectations were assumed. Given our result from Experiment 1 that listeners make use of both sensory and cognitive information in forming chord expectancy judgments, we would also expect sensory expectations to contribute towards listeners' pleasantness ratings.

#### (i) Sensory and cognitive expectations independently predict chord pleasantness ratings

To test the role of sensory expectations in musical pleasure, we fitted four Bayesian multilevel models. The first model predicted listeners’ pleasantness ratings of each chord based on the joint effect of IC and entropy from IDyOM, as in our original work. The second made predictions based on tonal dissimilarity as given by PP. The third was modified from the first by additionally including tonal dissimilarity as a predictor to model additive contributions of cognitive and sensory expectations. The fourth further included the interaction of IC, entropy and tonal dissimilarity to test for supra-additive effects.

**Figure 5.**
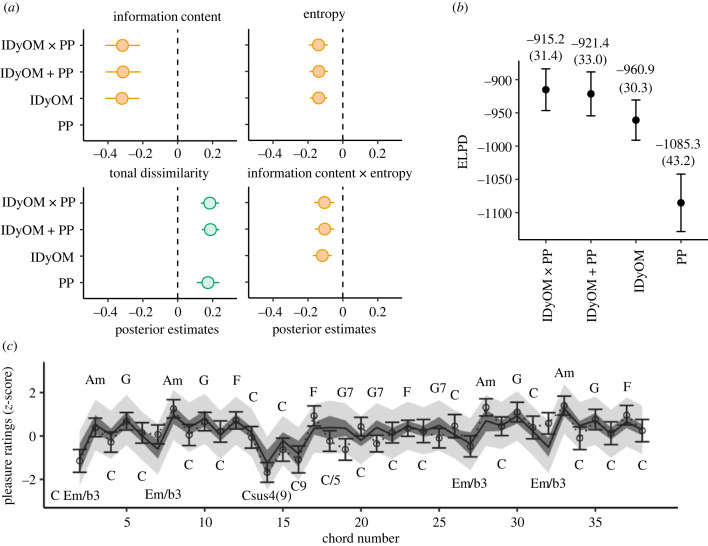
Sensory and cognitive expectations independently shape musical pleasure. (*a*) Posterior estimates (mean and 95%-credible interval) of standardized effects of expectancy simulations by PP and IDyOM remained virtually unchanged when simulations from the other computational model were introduced additively and supra-additively. (*b*) Incorporating simulations by both IDyOM and PP results in substantially improved predictive accuracy compared to a single model. Highly similar predictive performance between the additive and supra-additive model further suggests that the interaction term is redundant. (*c*) Predicting chord pleasantness responses with the additive model. Solid lines indicate the mean predicted pleasantness response to a chord progression derived from ‘Ob-La-Di-Ob-La-Da’ by The Beatles. Darker shaded regions show the 95%-credible interval of the mean. Lighter shaded regions show the 95%-credible interval of predicted responses. Open circles indicate actual mean behavioural response averaged across subjects. Error bars depict standard error of the mean. (Online version in colour.)

We first examined the out-of-sample predictive accuracy of the four models using cross-validation, as before. As shown in figure 5, the highest performance was achieved by the supra-additive model, closely followed by the additive model. However, that the expected gain was less than the standard error of the estimate (dELPD = 6.3, dSE = 6.7) again indicates overfitting in the supra-additive model. Nevertheless, substantial improvements in predictive accuracy were observed for the additive model relative to models based on simulations by IDyOM or PP only (dELPD = 39.5, dSE = 9.8, and dELPD = 163.9, dSE = 25.8, respectively). This indicates that we can best predict listeners' pleasantness ratings of chords unseen by the model when both sensory and cognitive expectations are considered.

Figure 5*a* also illustrates the fact that the standardized effect sizes of expectancy were almost identical in all four models. For the supra-additive, additive and IDyOM only models, the effect of IC (*β* = –0.317, 95%CrI = [–0.415, –0.216], *β* = –0.314, 95%CrI = [–0.412, –0.212], *β* = –0.319 and 95%CrI = [–0.416, –0.221], respectively), entropy (*β* = –0.140, 95%CrI = [–0.195, –0.086], *β* = –0.137, 95%CrI = [–0.191, –0.085] and *β* = –0.139, 95%CrI = [–0.189, –0.089], respectively) and their joint effects on pleasure (*β* = –0.106, 95%CrI = [–0.164, –0.049], *β* = –0.104, 95%CrI = [–0.161, –0.050] and *β* = –0.118, 95%CrI = [–0.174, –0.064], respectively) were highly similar and differed by an amount that was well within their 95% credible intervals. Likewise, for the supra-additive, additive and PP only models, the effect of chord tonal dissimilarity varied by at most 0.011 standard deviations (*β* = 0.184, 95%CrI = [0.134, 0.237], *β* = 0.188, 95%CrI = [0.139, 0.238] and *β* = 0.173, 95%CrI = [0.110, 0.239], respectively). That the effect sizes remained almost unchanged in the supra-additive, additive and IDyOM- or PP-only models suggests that cognitive and sensory information play complementary roles in predicting chord pleasantness. Furthermore, considering that the supra-additive effect of IC, entropy and tonal dissimilarity was essentially zero (*β* = 0.001, 95%CrI = [–0.051, 0.051]), these again indicate that expectancy simulations by IDyOM and PP each additively explain a unique part of listeners’ chord pleasantness ratings. It is therefore likely that sensory and cognitive expectations both shape musical pleasure—yet independently.

#### (ii) Contrary effects of cognitive and sensory surprise on pleasantness ratings

Finally, it is interesting that sensory and cognitive surprise could have opposing effects on musical pleasure—depending on the level of cognitive uncertainty. As seen in figure 5*a*, assuming that the entropy of a chord remained fixed at the average in the additive model, increased chord tonal dissimilarity was related to an increase in pleasantness ratings (*β* = 0.188]), whereas increased chord IC was related to a decrease in pleasantness (*β* = – 0.314). Nevertheless, the effect seemed to be larger for IC than tonal dissimilarity when comparing the magnitude of their effect sizes.

## 4. Discussion

Our study aimed to examine sensory and cognitive influences on harmonic expectations and aesthetic preference. Listeners gave continuous chord-wise behavioural ratings to chord progressions derived from commercially successful pop songs. We compared simulations from computational models of musical expectancy to ratings of surprise (Experiment 1) and pleasantness (Experiment 2). Four representative computational models along a sensory–cognitive continuum were considered: SD, PP, TE and IDyOM. We found that only PP and IDyOM could accurately predict chord surprise in musicians and non-musicians (with IDyOM explaining two to three times more variance than PP), and that they explained behavioural variance in surprise and pleasantness ratings in an independent and additive manner. These results support the view that sensory–acoustic information and acquired stylistic representations of music structure play complementary roles in modulating listeners' expectations—although with a larger contribution from the latter—which in turn, shape their enjoyment of music.

### (a) Evidence of sensory contributions to musical expectancy

Although the four computational models have been previously shown to model musical expectancy accurately, we did not find adequate evidence relating listeners’ chord surprise ratings and simulations from SD and TE. There are two plausible reasons for this. First, there are key differences between our continuous rating paradigm and the probe tone and priming studies for which SD and TE were validated. In this study, subjects gave surprise ratings for every chord as the stimulus was presented, whereas behavioural judgments in probe tone and priming studies were given only after stimulus presentation. This means that chord surprise ratings in the current study depended on a musical context that continuously varied as the stimulus progressed, whereas the context was fixed in length and often repeated in these studies on which SD and TE were validated. Second, our chord progression stimuli comprised chords sounded with a synthetic timbre combining a mix of marimba, jazz guitar and acoustic guitar, as well as a repeated drum sequence in the background. This is very different from the piano [31], violin [53], pure tone [35] timbres used in the probe tone and priming studies for which the model parameters were optimized. Furthermore, while TE has a higher-level component (the *tonal space*) that reflects similarity of tonal implications, it is still much less strongly cognitive than IDyOM as it lacks a concrete representation of chords depending on its preceding harmonic context. These arguments could explain both why SD and TE failed to generalize and the surprise ratings of non-final events in pop music chord progressions [10], or cadences in Mozart piano sonatas [34]. Computational models embodying sensory mechanisms may be particularly sensitive to such differences because they derive expectations from acoustic information in the input stimuli. Therefore, rather than arguing that listeners do not use mechanisms embodied by TE or SD in forming musical expectations, we take a more cautious stance and only suggest that these models were unable to generalize beyond their original stimulus setup and context to those used in the current experiment.

Nevertheless, we were able to accurately predict listeners' chord surprise ratings with PP, which indicates a significant sensory contribution to musical expectancy. This corroborates results from [40], which demonstrated that PP could replicate findings from 14 out of 18 existing behavioural and neurophysiological studies investigating harmonic expectations. Notably, that paper considered different parameter combinations of local and global pitch images to ensure that the replications were not dependent on parameter choice. In the current study, we took the same approach and considered six different parameter combinations for PP (see §2). We found that compared to the null model, incorporating simulations by PP substantially improved the predictive accuracy of out-of-sample chord surprise ratings for all parameter combinations (electronic supplementary material, figure S1). This not only highlights the generalizability of the model, but also provides robust evidence supporting the sensory influence of musical expectations. The results were also consistent with [51], in which PP was the only sensory model that had any predictive power on the neurophysiological response (P3a amplitude), but were inconsistent with [34], in which none of the sensory models had any predictive power. However, their null-findings could reflect the strong cognitive implications of cadential contexts, which render any sensory effects negligible by contrast.

### (b) Role of statistical learning in forming cognitive expectations

Our finding that IDyOM can accurately simulate chord surprise corroborates previous results with the same model [34,47,56]. It also provides further support for statistical learning as a plausible mechanism for internalizing regularities of a musical style, which are subsequently used to generate expectations. Harmonic expectancy as predicted by the tonal hierarchy is thought to be acquired through repeated and extended exposure to samples of Western tonal music and stored in long-term memory [27,39,56]. IDyOM extends this concept from zeroth-order to higher-order statistical regularities, allowing learning of more sophisticated stylistic regularities to be simulated.

Furthermore, simulations by IDyOM accounted for a larger portion of variance in chord surprise ratings compared to PP. This observation extends previous work in melodies [48] and provides further support for a larger contribution of cognitive over sensory information in forming musical expectations [31,35,43,46]. Given their increased musical training, the substantially larger difference in variance explained by IDyOM and PP by musicians compared to non-musicians further suggests that cognitive information is prioritized over sensory information when it is available. This interpretation could explain the facilitation of cognitive over sensory priming for rapidly presented chords when the stimuli had been previously presented at a slower tempo in [31]. We speculate that an expectancy mechanism that is applicable to a variety of contexts and is less reliant on sensory features would be favoured from a predictive coding point of view, as the ability to generalize increases the likelihood of minimizing long-term prediction errors [14–16].

One might suggest that the larger relative contribution by IDyOM could simply be due to a longer context integration window compared to the other three models. Based on a control analysis, we argue that this is not the case: We additionally trained a bigram variant of IDyOM that considered only a single chord as its context. This context length is comparable to the integration duration of our sensory models. We found that this bigram model still showed the best out-of-sample predictive accuracy and substantial improvement compared to PP (electronic supplementary material, figure S3), which indicates that a larger contribution of cognitive information was not driven by context length *per se*.

### (c) Sensory and cognitive information independently shape musical expectancy

Our finding that PP and IDyOM, which respectively embody sensory and cognitive mechanisms, can jointly improve predictive accuracy and explain independent variance in listeners’ chord surprise ratings strongly supports the proposition that musical expectancy is a function of both cognitive and sensory information. This result replicates previous work on melodies [46,48] and harmonic priming [43] but extends these findings to a comparison of strongly cognitive and strongly sensory models. This finding is also paralleled in language, where the intonation and pitch of speech [72–75] facilitate the inference of syntax. Our view is furthermore in line with the modularity hypothesis of music perception, which argues that music is processed in a distributed, parallel fashion [76,77] and is consistent with the PCM model, which posits that prediction errors are computed and propagated along all levels of the cortical hierarchy [14–17].

How might listeners combine cognitive and sensory information in generating predictions? Two possibilities have been proposed [48]. First, a single system learns and generates expectations based on both sources of information simultaneously. Second, two systems generate expectations based on sensory and cognitive information separately, which are then combined in a weighted fashion. Our data speak in favour of the latter, as we showed that the variance explained by IDyOM and PP was independent, non-overlapping and additive. This means that cognitive and sensory knowledge could be represented along two orthogonal dimensions without the need to represent the covariation between them. An auditory-specific system could generate expectations based on low-level sensory–acoustic features, while mechanisms such as statistical learning [78,79] or syntax processing [80] could compute expectancy as the auditory input is transformed into abstract representations such as chords.

### (d) Role of sensory expectations in musical pleasure

A key contribution of the current work is showing that listeners not only use sensory and cognitive information to form musical expectations, but that they also independently shape musical pleasure. This is in line with Huron's ITPRA framework of expectancy-driven musical pleasure [13,81], which postulates an immediate reaction response that is non-conscious and reflex-like, and a slow appraisal response that is cognitive and complex. It is moreover consistent with models of musical aesthetic judgment that divide the aesthetic experience in terms of an immediate sensory ‘core liking’ and a later ‘conscious liking’ response [82]. In terms of predictive coding, sensory ‘core liking’ would correspond to lower levels in the processing hierarchy, whereas the ‘conscious liking’ response would correspond to higher levels in the hierarchy [14,15]. PP could capture core liking, whereas IDyOM could be capturing conscious liking, particularly in musicians. Our finding that cognitive and sensory surprise could have opposite effects on listeners' chord pleasantness ratings in the current study further suggests that the two systems could work in concert in shaping musical pleasure. Interestingly, one study found that listeners gave similar preference ratings to musical stimuli presented in 750 ms or up to 1 min [83]. This suggests that aesthetic judgments were made primarily based on acoustic features before any musical relationships (such as chord progressions) could be integrated, and those judgments may have been updated and reinforced over time. However, it is important to distinguish between pleasure as modulated by static sensory effects such as timbre and consonance [84,85] (as postulated in [83]), from pleasure deriving from sensory expectations that require integration of acoustic features over time (which we study here).

The role of sensory features in shaping expectancy and pleasure as shown in the present work also highlights how music perception, and consequently the affective responses elicited by music, are inherently constrained by the structure of the human sensory system. It echoes the parallel between observing 1/*f* power-law distributions in rhythm and pitch in music across all human societies [3] and the increased sensitivity of sensory neurons towards signals that exhibit a 1/*f* structure [2], which also affects musical pleasure [86]. Such biological constraints could explain the recent finding that humans across multiple cultures show a preference for synchronicity in rhythm, but cultural-specific preferences towards isochronicity [87]. Whether cross-cultural consistency in the role of sensory influences on musical expectation persists in the face of cultural variability in cognitive expectations remains to be seen.

### (e) Limitations and future work

There are certain limitations in the current study. First, the conclusions we derived depend on our established link between chord ratings and simulations from computational models of musical expectancy. However, the extent to which each model could reflect human behaviour remains limited. For example, although we used PP and IDyOM to show the independent contribution of sensory surprise compared with cognitive surprise and uncertainty on chord pleasantness, we were not able to test the role of sensory uncertainty because PP does not have an explicit mechanism that models the uncertainty of an expectation. Furthermore, despite IDyOM's ability to accurately simulate listeners' chord surprise ratings, it is unlikely that they form chord expectations in the exact manner that is hypothesized by the model. As a variable-order Markov model, IDyOM assumes that the musical context is parsed serially and thus does not explicitly model dependencies between non-local musical elements. This is in contrast to the view that listeners parse music hierarchically with a representation beyond regular complexity and over multiple timescales [30,77,88,89]. It is also contrary to empirical evidence suggesting that listeners are sensitive to violations in long-range dependencies in music structure [90,91]. Future work could consider hierarchical models of musical expectancy following a model comparison approach of the current study. Although to our knowledge a working computational implementation is yet to appear, a promising candidate is the Generative Syntax of Music model [92].

It is also important to acknowledge that the chord progression stimuli in the current study were derived from commercially successful pop songs in the Western tonal tradition. Although this provides a high degree of ecological validity, it implies that only a subset of possible chord progression combinations has been presented, and the extent to which a chord is surprising or pleasant is only relative to other chords found in this musical style. Previous studies have shown that the same chord in a progression could evoke different expectancy and preference ratings depending on whether the stimuli were composed in the style of common-practice or rock music [45,93]. These results highlight the role of stylistic context in shaping perception and consequent emotional response during music listening, and therefore suggest the need to demonstrate that the current findings generalize to other musical styles. Evidence suggests that statistical learning of music structure could be implicit [94], and rapid [38], and also observed in other musical styles [95,96].

Another interesting question that remains is how repeated listening integrates with an expectancy-driven mechanism of musical pleasure. Listening to the same musical stimuli repeatedly has been related to both increase [97,98] and decrease [98,99] in liking, and has been shown to attenuate a neurophysiological marker that is evoked from harmonically surprising chords in a progression [100]. One hypothesis from the current experiment is that sensory surprise remains unchanged after repeated listening over long timescales (e.g. once a week), but is attenuated after immediate repetition due to adaptation effects. Uncovering how and over what timescale stimulus repetition shapes cognitive and sensory expectations would be a crucial next step.

Finally, while the current study demonstrated the independent contribution of cognitive and sensory expectations in shaping musical pleasure, future work needs to clarify the precise mechanisms relating expectancy, pleasure and the aesthetic experience [101]. From a PCM perspective, music perception is an act of ‘active inference’ as listeners deploy attention towards resolving hypotheses for musical events in the upcoming musical passage [14,15]. In line with the Learning Progress Hypothesis [102], music could confer reward value by continuously providing opportunities to learn and improve listeners' internal model for future predictions [15]. While plausible, empirical evidence remains to be demonstrated, and it is still unclear how a learning account fits into models of music engagement, which argue for transitions between attentive and mind-wandering states during music-listening [103]. Resolving these questions would prove fruitful towards understanding our timeless appeal for complex structured auditory sequences also known as *music*.

## Data Availability

The stimuli, code and datasets analysed during the current study are available from the OSF repository: https://osf.io/5fk2q/ [104]. Supplementary material is available online [105].
